# On the brink of social resistance: local community perceptions of mining company operating permits in East Luwu, Indonesia

**DOI:** 10.3389/fsoc.2024.1373736

**Published:** 2024-10-17

**Authors:** Sawedi Muhammad, Suryanto Arifin, Ridwan Syam, Bama Andika Putra

**Affiliations:** ^1^Department of Sociology, Universitas Hasanuddin, Makassar, Indonesia; ^2^School of Sociology, Politics, and International Studies, University of Bristol, Bristol, United Kingdom; ^3^Department of International Relations, Universitas Hasanuddin, Makassar, Indonesia

**Keywords:** mining companies, local community perceptions, mining licensing, social resistance, corporate social responsibility

## Abstract

The presence of a mining company in an area affects the living conditions of the surrounding community from an environmental, social, and economic perspective. This research aims to contribute to the literature on social perceptions concerning mining company operations by assessing the local societies’ rate of rejection or acceptance of a mining company’s extension of operating permit, along with reasons that justify the decision. We conducted semi-structured interviews with seven interest groups in four sub-districts in East Luwu Regency, comprising 79 participants. This research uses a combination of quantitative data collection and in-depth qualitative interviews to strengthen the sources’ reasons for providing answers. The results show a high rejection rate expressed by communities living in the sub-districts where mining companies operate, alongside a strong preference for extending operating permits for sites that are of greater distance to the smelters. If the business permit is ultimately transferred to the existing company, it may exacerbate community tensions and resistance. In that case, the local community wants several priority improvements, namely, increasing the number of workers working in the factory, further boosting economic growth and empowerment programs carried out in affected areas, and improving post-mining environmental management. Therefore, this study contributes to the discourse of local community perceptions in mining areas, which may affect mining operations in the future.

## 1 Introduction: mining companies and social perceptions

The presence of a mining company in an area influences the living conditions of the surrounding community in environmental, social, and economic aspects. To minimize environmental losses, a mining company must complete an environmental impact analysis document because non-compliance with social and environmental requirements will result in high costs for the mining company and thus can jeopardize the feasibility of mining operations (Kemp and Owen, 2013). It’s just that affected communities in mining areas still often face challenges such as increasing numbers of immigrants, increasing difficulty in owning a home, and inflation (Moffat and Zhang, 2014; Dikgwatlhe and Mulenga, 2023), and also have an impact on land, water, and food security for local communities (Obeng et al., 2019; Blanco et al., 2023). Therefore, apart from having obtained a Mining Business Permit (IUP) from the government, the mining industry must also ensure that its operations align with the practices of attaining a social permit to operate from affected communities (Black, 2017; Boutilier and Thomson, 2019).

Social permission to operate is related to the acceptance and approval of affected communities for mining activities in their area. The community provides this with a transparent relationship between the company and the community (Moffat and Zhang, 2014; Black, 2017; Boutilier and Thomson, 2019). The permit is valid when the community is satisfied with the company’s socialization or plans for mining activities to be carried out. Without social permits, community resistance or rejection of the company in detrimental forms will emerge (Franks et al., 2014; Moffat and Zhang, 2014; Boutilier and Thomson, 2019) and can even stop mining operations that are already underway altogether (Franks et al., 2014; Lèbre et al., 2021). A social permit to operate is not written or stated in an official document. This is an agreement given by different stakeholders, namely the affected community as the main stakeholder, and also other groups such as government, organizations, media, religious institutions, NGOs, and other companies that are appropriate to this context (Freeman et al., 2004; Mutti et al., 2012; Black, 2017).

Previous studies examined the social license to operate in various mining cases in different parts of the world. Studies conducted in Australia and Brazil showed that social licenses to operate depend on the social contexts in which operations are planned to take place (Hall et al., 2013; Moffat and Zhang, 2014; Gaviria, 2015). Social license to operate emerged in the mid-1990s from within the mining industry as a response to social risks (Boutilier and Thomson, 2019). Furthermore, the concept has been used by various actors in the resources sector, including mining companies, civil society and non-governmental organizations, research institutions, governments, and consultants (Kurlander, 2001; Slack, 2009; McNab et al., 2013; Black, 2017). The social license to operate has also been implemented and adapted by various other industries, including manufacturing and paper, alternative energy generation, and agriculture (Gunningham et al., 2004; Williams and Martin, 2011; Hall et al., 2013). Not much has been revealed about the basis for the affected community to grant their social license. Apart from that, revealing the aspects considered valuable by the affected communities for continuing mining operation permits is interesting.

In Indonesia, precisely in mining areas, there have been many acts of resistance from communities living around mines with certain companies, both because of economic problems, the value of mining for the community, and long-standing conflicts related to customary land (Erb, 2016; Libassi, 2024; Bakker, 2023). Local community demands regarding the company’s mining activities are due to the community not being given the attention it deserves concerning society’s economic, social, and environmental concerns (Coulson et al., 2017). This is also linked to conflicts between local communities and national and multinational mining companies due to social aspects, environmental problems, and the economic impact of mining. A concerning development has also been the tendency of mining companies to expand their mining area to an area cultivated by the community for years in gardening or farming.

Nevertheless, resistance to mining companies is only one discourse out of many in the context of Indonesian mining operations. In the past decade, Indonesian scholars have highlighted the impact of mining operations in diverse regions of Indonesia, outlining what the community perceives as pivotal for continuing its operations in the future. Research has delved into assessing the impact and perceptions toward limestone, gold, coal, and tin mining, focusing on different aspects. For example, Awandoi and Biraputra assessed the community perceptions toward the limestone mining taking place in Papua, with positive outlooks due to the company’s consideration of local customs (Awandoi and Birawaputra, 2018). For gold mining in Bima and Buru Regencies, scholars have been in consensus that the mining companies operating in those areas have not fulfilled expectations toward environmental protection and the provision of economic opportunities for locals in affected areas (Khosiah et al., 2017; Sehol et al., 2022). Meanwhile, on the topic of coal mining in Indonesia, there has been a consensus among scholars in which the dominant argument has been the presence of expectations so that empowerment programs consider the impact of mining operations on the environment and possible respiratory diseases that may occur (Asfawi and Isworo, 2023).

For South Sulawesi, there have been conflicting views toward mining operations operating throughout the region. Most have continued past discourses on how society demands mining companies consider the environmental impacts (Syarifuddin, 2022; Agussalim et al., 2023). However, a rising number of studies have highlighted the different opinions held within society. For Adnan and Somantri, the contrasting voices within society lead to social conflict, with several parts of the society demanding the continuation of operations while others being critical and rejecting its continued operations (Adnan and Somantri, 2022). In a recently published article, Sawedi and several scholars argued the importance of looking into different stakeholder interests to better understand why social conflicts occur and what each group considers pivotal in the operation of mining companies in Sulawesi. They argued that government elites, community leaders, women figures, local workers, and mining company employees all reserve different thoughts due to their different interest levels (Muhammad et al., 2024).

The existing discourses on mining companies in Indonesia still lack empirical investigation of diverse cases, especially in Sulawesi. With different mining operations in the region, an investigation of mining companies in Sulawesi allows the advancement of existing literature on the lens through which the society affected in mining operating areas perceives such operations. Furthermore, no research has been conducted specifically targeting affected communities based on villages in which mining company facilities are located, and those located in a more distant location fall under the scope of the company’s empowerment programs.

This research aims to contribute to adding to the literature on the social license to operate mining companies through an empirical investigation in Indonesia. We intend to reveal quantitatively and qualitatively the level of social permission from the company ‘Vale Indonesia’ (hereinafter referred to as Vale) to operate in East Luwu Regency, which is currently in the process of obtaining a business permit from the Indonesian government. In doing so, four sub-districts are assessed. The first sub-district is located in Nuha, and the location of Vale’s smelters. Meanwhile, the other three sub-districts of Towuti, Wasuponda, and Malili, located in close proximity to the smelters (thus, a target area of Vale’s corporate social responsibility (CSR) programs). This article also aims to answer the question regarding what aspects are considered necessary by the affected communities that need to be improved if the company obtains a business permit and social permit. Finally, the worst condition was revealed if the company had to be replaced, including which party the community considered appropriate to continue the nickel mining project in their area.

## 2 Method

This research uses a combination of qualitative and quantitative data methods. We conducted semi-structured interviews with seven interest groups in four sub-districts in East Luwu Regency, namely Nuha, Towuti, Malili, and Wasuponda. These interest groups include government officials, women’s leaders, fishermen or farmers, employees, formal community leaders, traditional leaders, and local entrepreneurs.

As many as 79 participants were interviewed, using quantitative data collection and qualitative in-depth interviews to strengthen the speakers’ reasons for providing answers. For the in-depth interviews, open-ended questions in relation to their acceptance or rejection of an extended contract for the mining company in operation, thoughts about areas of improvement, and potential replacement stakeholders were asked for approximately 20–40 min. The participants were randomly approached as long as they resided in the four sub-districts determined in this study and fell under the category of the defined interest groups. Everyone speaks the same official language, namely Indonesian. Data collection took place in March 2023. This timeframe was critical, as Vale was in the process of extending its license permits, and a deeper understanding of the local perceptions was critical to the success or failure of the extension. We guarantee the anonymity of our interviewees per Hasanuddin University research ethics standards, where researchers and resource persons are not required to sign a written agreement, as they only agree verbally to participate.

The interviews with some participants took place in their homes and workplaces. Individuals were asked to answer a questionnaire with dimensions of approval for the extension of the company’s license, aspects of corporate social responsibility that need to be improved, and parties that are appropriate to replace the current company. Descriptive analysis was carried out to see the level of participant perception, and it was complemented by information from interviews. Meanwhile, the interview transcripts were analyzed by identifying the dominant discourses that emerged within society.

## 3 Results and discussion

Understanding the perceptions of local communities around the mine regarding the existence of mining companies in their area plays a vital role in assessing the knowledge these communities possess. This is because the nickel mine in the East Luwu Regency area has been operating for approximately 50 years. In 2023, the permit for the company’s continued operations is being extended, as the company’s work contract with the Indonesian government will end in 2025.

This section presents key research findings, including the perceptions of local communities in four mining-affected areas in East Luwu Regency, Indonesia. Namely Malili District, Wasuponda District, Towuti District, and Nuha District. The local community in question comprises local people from various levels of groups who are interested in the company’s existence.

### 3.1 Respondent’s demographics

The demographic profile of respondents greatly influences individual perceptions, especially in mining areas, and is an essential factor in perception studies. For this reason, related demographic variables, such as gender, age, educational status, and employment status, were measured for those participating in this study (Table 1).

**Table 1:** Demographic profile of respondents.

Demographic element of respondents	Description
Gender	57 men (17 Malili, 16 Wasuponda, 9 Nuha, 15 Towuti)
	22 women (3 Malili, 3 Wasuponda, 12 Nuha, 4 Towuti)
Age (years)	21–25 (2 respondents)
	26–35 (12 respondents)
	36–45 (22 respondents)
	46–55 (25 respondents)
	56–65 (12 respondents)
	More than 65 (6 respondents)
Education background	Not completed primary school (2 respondents)
	Completed primary school (19 respondents)
	Completed junior high school (11 respondents)
	Completed senior high school (31 respondents)
	Completed a diploma degree (3 respondents)
	Completed undergraduate studies (13 respondents)
Category of work	Agriculture, forestry, fisheries (36 respondents)
	Mining (9 respondents)
	Processing industry (2 respondents)
	Construction (1 respondent)
	Wholesale and retail trade (3 respondents)
	Government administration (7 respondents)
	Health and social worker (2 respondents)
	Other services (19 respondents)

### 3.2 Public perception over the continuity of company operation

Two questions were asked through a semi-structured questionnaire to obtain respondents’ responses on the issue of extending mining company operating permits in their area. The first question is based on the respondent’s perception regarding support for continuing. The following question was raised, “Do you agree that the Vale Company will continue in 2025?” to determine the public’s opinion regarding public approval about the continuation of the company’s permit. The next question was asked regarding why respondents agreed or disagreed with the company continuing to operate. What considerations are considered when giving support, and what reasons are held for the contrasting opinion?

**Figure 1:**
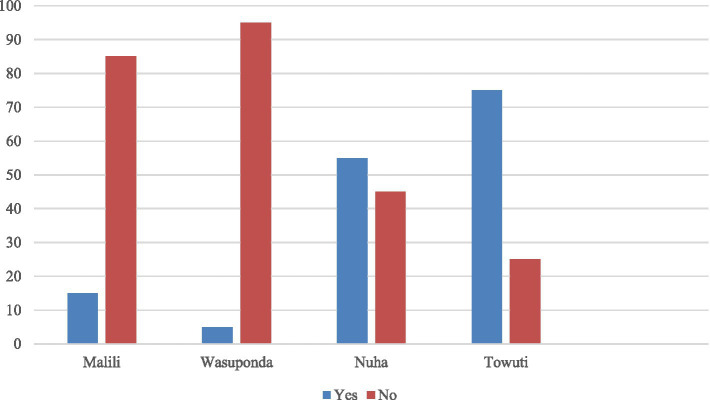
Community perceptions regarding continuing social license to operate (percentage). The total participants: Malili (20 respondents), Wasuponda (19 respondents), Nuha (21 respondents), and Towuti (19 respondents).

In Figure 1, it can be seen that there seem to be regions that show a high rate of rejection, while others display low or even between rejection and acceptance. If we look at the public’s perception of each region, only two regions support the company’s continued operation permit (Nuha and Towuti). Meanwhile, community groups prefer not to continue in the two other affected sub-districts. Interestingly, the two sub-districts that refused were areas where the smelter was located (Malili and Wasuponda). This means that local community groups in the areas closest to the factory prefer that the company not continue with its operating permit. At the same time, this opinion is not shared for those located more distance from the smelters.

The interviews showed a higher expectation shared among locals from the interest groups in the nickel smelters (Malili and Wasuponda). Due to this expectation and the failure of Vale to provide sufficient benefits, the dominant opinion has been to reject the extension of Vale operating within their sub-districts. For Nuha and Towuti, areas of proximity to the mining operations, there is a favorable response due to the additional benefits Vale provides compared to if it were absent. One thing to note, however, is that even in Nuha and Towuti, the level of rejection is also high, making an investigation of their perceptions intriguing.

The informants supported continuing the mining company’s current operating permits based on various considerations. This includes consideration of regional income for continued development in the region and multiple programs from which the community has felt the benefits. They do not want the running program interrupted if the company’s permission is not continued. As stated by the following ‘NS’ informant:

*I agree with permission for Vale to continue in Sorowako. Because without Vale, East Luwu would not be like this. The source of PAD from Vale is more than 300 billion, and it looks like 370 billion every year. Another thing is scholarships for school children and the conduct of various development programs for the community* (‘NS’ Informant, 09 March 2023).

From the information conveyed by informants, community interest in development progress in East Luwu is one of the considerations for supporting the continuation of mining companies in the area. This means that the community considers that with the support of nickel mining, which has been operating in their area, the community will experience various developments by the government. The East Luwu Regional Government has received significant revenues from nickel mining. As in the 2022 Regional Development Work Plan (RKPD) in East Luwu Regency, there are six priorities for using the budget for development, including (1) improving education and health services, (2) fulfilling infrastructure and facilities for the agricultural and tourism sectors, (3) strengthening infrastructure to support the agricultural sector and tourism, (4) increasing investment and competitive business opportunities, (5) realizing good and innovative governance, and (6) maintaining religious tolerance and preserving cultural values.

Apart from that, the opinion that supports the continuation of the company’s permit is also based on doubts about the government’s ability to take over management of the mine. Informants believe that managing this nickel mine requires high costs, and they are unsure that the government can manage it. This is confirmed through the following statement from Informant’ NA:’

*The important thing is to be able to manage it because this is a big company, and that’s what you need to think about. If the government decides not to extend Vale’s contract, are they capable of what Vale has done so far? Otherwise, it will be detrimental to society. Can it run at the level at least what Vale has achieved today, including wages? If you want to be empowered by regional contractors, that would be good, but can we afford it? We know the standards Vale adopts are high as it is a foreign company* (‘NA’ informant, 09 March 2023).

The public perceives that management based on foreign company standards is still much more convincing than if the government managed it. There is still an element of worry about facing change because they have experienced various corporate social responsibility-related facilities during the mining company’s operations, starting from the previous mining company Inco to the present Vale.

The continuation of the company permit was also supported by other informants, considering that the company has significantly impacted society, including improving the economy. As stated by informant ‘AB’ below:

*We cannot deny that Vale has improved the community’s economy. That’s why we defend Vale and want Vale to stay here when we meet. CSR and community empowerment must be fixed before the contract continues* (‘AB’ Informant, 09 March 2023).

Mining is accepted to operate in the community with the hope of providing benefits to companies and local governments and impacting the welfare of local communities. The community has perceived mining activities in East Luwu for approximately 50 years as having improved the community’s economy. According to data from the Central Statistics Agency (BPS) for East Luwu Regency in 2022, regional economic growth in the region has grown by 1.99%. However, in 2021, it experienced a decline of 1.39%. The cause of the decline in the previous year was triggered by the mining sector, which is East Luwu’s flagship, experiencing a decline in production and other operational-related aspects. This shows that mining in East Luwu has dramatically impacted economic growth rates in the region.

Nevertheless, there have also been a considerable number of rejections concerning the plan for Vale’s contract extension in the region. Other informants expressed different views, stating that they did not agree or did not want the company to be granted an operating extension permit at this time. What is considered the company’s current failure is related to the involvement of local contractors. As stated by the ‘BU’ informant during the interview:

*I consider the distribution of projects to local contractors to be uneven. Vale does not empower local contractors. If Vale has work to do, it should be given first to local contractors who can manage it, then to national contractors. Hundreds of local contractors are in this empowerment area (four sub-districts)* (‘BU’ Informant, 09 March 2023).

The informant’s statement revealed that the reason for not agreeing to continue the company’s operating permit was related to local contractors’ interests in obtaining work projects from Vale as the management company. As has been going on so far, Vale carries out mining business activities following the Law of the Republic of Indonesia No. 3 of 2020, which requires prioritizing local contractors and workers. For this reason, Vale has always attempted to engage with national and local contractors. It’s just that the informant perceives that the company has not optimized local contractors so far. Meanwhile, approximately 100 local contractors are already ready to accept projects from Vale in the four sub-districts, which are classified as empowerment areas.

In addition to the issue perceived as pivotal by local contractors, another reason other informants refuse to continue the mining operation permit relates to Vale’s CSR programs. This was revealed from the following ‘SU’ informant’s statement:

*CSR is the community’s right to obtain the impact of the company’s activities. The company is obliged to assist, but we do not know how much concentration it gives or how much profit it makes. This means that if we had heard that yesterday, around two and a half percent of the company’s profits would have been raised. However, in East Luwu, not all of the 15 sub-districts are in the empowerment area; only four are in the empowerment area. Malili alone, out of 14 villages of 1 subdistrict, only nine villages can get it, namely Puncak Indah Village* (‘SU’ informant, Interview 10 March 2023).

From the informant’s explanation, it is known that the reason for refusing to extend the permit from the company at this time is dissatisfaction regarding the implementation of the CSR program. Vale continues to manage the mine as well as its CSR from the previous mining company, Inco, which empowered four sub-district areas, namely Malili District (nine villages and one sub-district), Wasuponda sub-district (six villages), Towuti sub-district (18 villages), and Nuha sub-district (four villages, one sub-district). Community empowerment programs as a form of company CSR in the social sector include coaching programs, mentoring, and assistance with production equipment. This slightly differs from Inco. However, a problem for the community is that the coverage of this company’s empowerment area did not increase for five decades until 2013. Namely, in four sub-districts, even in Malili Sub-district, out of 14 villages/sub-districts, only 10 villages/sub-districts have been included in corporate empowerment.

### 3.3 Public perceptions on the aspects that require improvement

**Figure 2:**
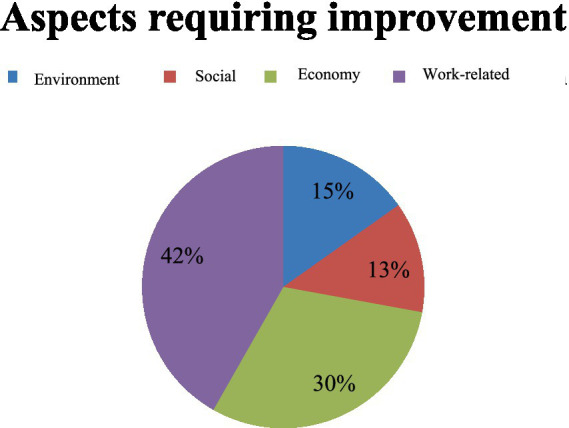
Public perceptions on the aspects that require improvement.

In this second problem formulation, it is assumed that the government continues the company’s business license. Following up on this, information was collected from respondents regarding the most critical aspects for the company to improve in the continuation of the next contract. The elements in question relate to environmental, social, economic, and employment. Respondents’ answers can be seen in Figure 2.

The local community considers several aspects of implementing the CSR program that the company has carried out so far, but it still needs improvements. Firstly, what needs the most improvement is the involvement of local workers to work as employees in factories with local or national contractor status and to have their working status elevated to become permanent employees of Vale. Second is an economic improvement, in which even though in 2022, according to BPS data, the mining sector has contributed to economic growth in East Luwu, the community believes there is still a need for improvement in community empowerment activities. So, if the mines close, this can be an anticipatory action for local communities that still have economic independence.

Third, in the environmental management aspect, the community considers the company’s management efforts to preserve the pre- and post-mining environment in East Luwu to be quite good compared to mines in other areas. For example, as stated in ‘Katadata.co.id’ in September 2022, Vale received the ‘2022 Good Mining Practice Award’ for the third time. Therefore, according to Figure 2, the community assesses that environmental aspects are in the third aspect position necessary for future program improvement. Even though it is commendable, mining activities certainly impact the environment, such as deforestation, water pollution, decreased air quality, and the threat of landslides. If the mining operation permits continue, the community views this as needing improvement for the next period.

Fourth, regarding social aspects. The public considers this to be the last of the other aspects the company can improve. Based on the informant’s explanation, during the 54 years the company has been operating, many types of social programs have been carried out. Starting from social assistance in the form of cash assistance to community empowerment programs. It’s just that programs are still changing. Therefore, the community has not yet seen the existence of an empowerment program that can significantly impact local communities.

These findings show that the public still wants the presence of companies provided there are improvements, especially in the economic aspect. Regarding theoretical terminologies, this is known as economic legitimacy (Darling, 2011), namely when stakeholders assume that the company provides benefits for them. It is perceived that the company always contributes to the welfare of the area around the mining area, fulfills community expectations with its social role, and shows justice.

In alignment with the existing literature, unsurprisingly, there is a clear pattern between the proximity of the mining operations and expectations of the areas of improvement. The dominant requests for improvement are related to work, which is voiced mostly by interest groups close to the mining operations (Malili and Wasuponda). Meanwhile, in Nuha and Towuti, most have expressed more secondary concerns in their expectations, focused on the social aspects.

Several informants in Malili and Wasuponda expressed issues with Vale’s approach to local workers in the sub-district. The dominant discourses have focused on the lack of consideration toward local contractors, the use of local workers, and the minimum number of skill-developing programs. For those locals, internal regulation from Vale needs to consider the disparity between national and local contractors and the difficulty of competing for those living in villages. As several of the following informants have expressed:

*“Local contractors need to be empowered. Although there are several regulations that discuss this, local contractors need to be able to feel the direct benefits from the mining companies. Even though there is already a favoring of local contractors, the reality is that this has not been the case”* (‘BU’ informant, Interview 10 March 2023).

*“Opening tenders nationally is the problem for us. Sometimes, the requirements are just not possible for us to fulfill. For example, a minimal amount of capital and assets is required in several cases. There needs to be a regulation that bridges between national and local contractors. But these regulations need to be careful so that it is not the national contractors that benefit most by becoming the main stakeholder, while local contractors have limited functions in the partnership”* (‘OU’ informant, Interview 09 March 2023).

*“Mining companies need to give the opportunities to local workers. If local workers can work on projects, mining companies should not prioritize those located not from the region. Local workers first, except for works that our local workforces cannot do, for example, not enough qualification, then national workers can be included.”* (‘FD’ informant, Interview 09 March 2023).

On the topic of skill-developing programs, locals in the Malili and Wasuponda sub-districts discussed the connection between the lack of local tenders being granted projects by Vale and the lack of skill-development programs offered. In explaining their expectations for the future, some have also referenced the programs operated by the previous mining company operating in those regions, ‘Inco,’ due to their inclusive approach in the surrounding mining areas. The areas in which Vale is currently located are villages that have limited human resource qualities compared to other parts of the nation. Therefore, it was unsurprising to hear several respondents expressing their concerns. One of them was expressed by the informant ‘BU:’

*“Empowerment towards the community needs to be increased in the future. The focus should be on advancing skills in society, not just mere workshops. The key should not be that these programs are operated just to tick a checklist, but to provide clear impacts towards the people”* (‘BU’ informant, Interview 10 March 2023).

Meanwhile, Nuha and Towuti locals believe that these mining companies can be more involved within the community in the future by advancing more funding opportunities and allowing locals to develop independent economic opportunities that are sustainable. In expressing their concerns for the future, many also referenced ‘Inco’ and how the future mining company operating in the region must be able to match the programs once operating in their sub-districts. Informant ‘MT,’ for example, expressed how the company benefits (education and health services) were only given to those directly employed with Vale (or a family member):

*“[on the question of whether the informant attains scholarship from Vale] … For that, it is only for employers. Those employed would have their education paid in full”* (‘MT’ informant, Interview 09 March 2023).

Others, such as informant ‘SI,’ expressed how, as an art trainer, Vale has not shown any support through its CSR programs for the past couple of years. She expressed her sadness that Vale seemed to place a closed eye on social aspects within the community and their failure to support any of her initiatives. This could be a valuable input for company policymakers in the mining industry.

### 3.4 Public perception regarding the appropriate party to replace the current company

In regards to the discussion of a possible replacement for the company, there is no decisive answer based on the close or distanced proximity to the mining operations. Respondents from Malili, Waspudonda, Nuha, and Towuti are not concerned about the company potentially replacing Vale. The priority of the respondents is to ensure that whether it is a private or government-owned entity, the requested improvements need to occur. Nevertheless, there is evidently clear skepticism that government-owned companies can supersede Vale’s performance. One of the respondents, ‘FD,’ expressed this:

*“Vale, state-owned enterprise, or local-owned enterprise does not matter. Even the local-owned enterprises planned for East Luwu five, have not been operating until now. This is a problem, considering that our region’s budget allocation is high. Suppose the government decides to cancel the permits and take responsibility for the mining operations. In that case, the government needs to prepare a considerable number of capital, which may not be able to be done. So anything is possible, but the key is that the stakeholder must be able to continue this difficult operation, and needs to think thoroughly whether it can continue the success made or not”* (‘FD’ informant, Interview 09 March 2023).

Expressing that Vale’s capacity as a mining company is average, the informant ‘BU’ expressed that Vale is easily replicable compared to other companies in Indonesia, but Vale does not have much to show for it. The informant stated that:

*“Basically, Vale is not much compared to other big national companies out there in Indonesia. However, because Vale is a corporation that builds its foundation by itself and is self-sustaining, people perceive it as a major company in the nation. For example, if we compare it with one of the nation’s private cigarette companies, we will see a high disparity between the companies.”* (‘BU’ informant, Interview 10 March 2023).

Corresponding to the negotiation process with government officials, there is the possibility that Vale Indonesia’s contract would be terminated. This means that this mining company from Brazil may have to end its activities and abandon the nickel mine in East Luwu if a permit to continue operations is not obtained before 2025. Therefore, in this section, community perceptions regarding parties deemed appropriate or accepted by the local community are revealed as an alternative replacement for the company. Alternative Mining Business Actors that have the capacity to replace the management of nickel mines in East Luwu are foreign companies that are equivalent to Vale, which includes a private company operating in the mining sector, a State-Owned Enterprise (BUMN), or a Citizens’ Cooperative (BUMD). The answer options that were offered to respondents were for those who were resistant to mining activities.

Most respondents chose BUMN as the party they believe can replace the current management company. Several state-owned companies in Indonesia operate in the mining sector, such as Bukit Asam (coal), Antam (gold), Pertamina, Perum Gas Negara, Tin, and other state-owned companies. The community assesses that BUMN’s performance in the mining sector can replace Vale if the permit is not continued. Furthermore, the second party considered to be able to replace Vale is another foreign company. This means that the public perceives that the nickel mine in East Luwu must be managed with a large budget so that the appropriate party is still a foreign company. Apart from that, foreign companies are considered to be able to provide outstanding CSR for the surrounding community.

Meanwhile, 11.4% of respondents thought mining should be banned because it damages nature. This means that people who refuse to have high environmental awareness choose to obtain prosperity by relying on sources of livelihood other than mining. This corresponds to a study in 2009, which echoed the importance of local communies knowing their environment and the issues that affect their welfare (Dogaru et al., 2009).

Local communities are part of the stakeholders or groups directly and potentially impacted by mining management. Therefore, analyzing local community perceptions is essential for continuing mining operation permits in their area. Based on the data found, it is known that the perception of local communities around the mine shows different responses between those who agree to allow the company’s mining operations to continue and those who disagree or refuse to continue. There are differences in views, which are also influenced by regional aspects. Some predominantly support certain sub-districts, but other sub-districts, even those closest to the factory or smelter, reject it. Socio-demographic factors, such as age, education, and political ideology, significantly influence perceptions, views, awareness, attitudes, and concern for specific environmental issues and risks (Dogaru et al., 2009).

From the informants’ statements, it is known that there are four reasons that local communities are concerned about the mining permit to continue. Consideration of support for mining operation permits is continued due to (1) the company’s assistance, which is considered significant to the regional government; (2) the continuation of the social assistance program; (3) distrust in the capabilities of government-owned companies; and (4) the continuation of the community’s economy. Generally, the dominant respondents’ considerations relate to the continuous support of the company’s compensation to the government and community. This confirms the findings made by Owasu-koranteng in his assessment of the community struggles faced vis-à-vis mining activities in Ghana. He explained that most of the compensation given to communities in mining areas is provided to them by large-scale mining companies (Owusu-Koranteng, 2008). This will effectively reduce the threat of community decisions to refuse permits to continue mining.

From the informants’ statements, it is known that there are four reasons that local communities are concerned about the mining permit to continue. Consideration of support for mining operation permits is continued due to (1) the company’s assistance, which is considered large to the regional government; (2) the continuation of the social assistance program; (3) distrust in the capabilities of government-owned companies; and (4) the continuation of the community’s economy. In general, the respondents’ considerations as the dominant local community support the continuation of the mine because of the considerable compensation that the company has provided to the government and community. This point also confirms the findings made by Owasu-Koranteng’s study, which explains that most of the compensation given to communities in mining areas is provided to them by large-scale mining companies (Owusu-Koranteng, 2008). This will effectively reduce the threat of community decisions to refuse permits to continue mining.

Meanwhile, opinions that reject the continuation of mining business permits are based on two considerations: the lack of empowerment of local contractors and the small number of areas that receive the impact of the company’s CSR. The two aspects of refusal from respondents are related to the welfare of the surrounding community. It is hoped that the presence of multinational mining companies collaborating with national contractors will enable local communities who form local contractors to be involved by the company. These findings reinforce the findings of Harvey and Brereton that public and community expectations of mineral companies are increasing (Harvey and Brereton, 2005). Moreover, this corresponds with the increasingly developing discourse on CSR and sustainable development.

## 4 Conclusion

The expiration of the company’s work contract and the entry of an extension period in the form of an operational permit raises pros and cons for local communities in the mining area in East Luwu Regency. Our findings show that the company’s performance so far has convinced local communities in the mining area to predominantly continue to provide support for the company’s continuation now that it can continue. Despite improving program interventions in the two nearest sub-districts, refusal remains. The considerations for supporting the company’s permit to continue are due to the immense value of the company’s assistance to the regional government, the continuation of the social assistance program, doubts about government-owned companies, and the community’s economic continuity, which is perceived to have increased with the current management of the mining company. Meanwhile, the rejection was due to the treatment of local contractors and the lack of expansion of empowerment areas that received the company’s CSR program, so it did not meet the local community’s expectations.

Suppose it turns out that mining business permits are continued with existing companies. In that case, the local community wants several priority improvements, namely increasing factory worker numbers, economic growth, and empowerment programs carried out in affected areas, and improving environmental management post-mining. Meanwhile, if it turns out that the government is not continuing the business permit for the current company, local people would prefer that a government-owned company, namely BUMN, take over the mining operations or continue to use a foreign company with management capabilities that are on average the same as existing companies.

Even though the position of local communities in providing social permission for the continuation of company operations is still weak, we think this is still important for decision-makers. The government needs to consider the community’s views as the closest parties who feel the impact of mining and companies.

The limitation of this research is that it only contributes to the involvement of participants with a relatively small percentage and involves stakeholders who are not yet comprehensive. Therefore, this research suggests further research with a broader range of participants to produce more accurate data regarding local community perceptions. Apart from that, further research is needed to objectively measure the performance of companies in mining areas in various scientific disciplines to obtain a comprehensive analysis of the impact of mining on society and the environment.

## Data Availability

The raw data supporting the conclusions of this article will be made available by the authors, without undue reservation.
